# Grape Pomace as a Platform for Secondary Metabolite Recovery: Mechanistic Insights into Bioactivity, Processing, and Functional Valorization

**DOI:** 10.3390/foods15091531

**Published:** 2026-04-28

**Authors:** Monica Trif, Claudia Terezia Socol, Mihai Domnuțiu Suciu, Alexandru Vasile Rusu, Florin Leontin Criste, Daria Rus, Constantin Bîtea, Mohammad Billal Hossain, Lubna Ahmed

**Affiliations:** 1 Institute of Life Sciences, University of Agricultural Sciences and Veterinary Medicine Cluj-Napoca, Manastur 3–5, 400372 Cluj-Napoca, Romania; monica_trif@hotmail.com; 2 Department of Animal Science and Technology, University of Oradea, 1 University St., 410087 Oradea, Romania; clausocol@yahoo.com (C.T.S.); florinleontincriste@gmail.com (F.L.C.); cbitea991@gmail.com (C.B.); 3 Department of Urology, Faculty of Medicine, “Iuliu Hatieganu” University of Medicine and Pharmacy, 400012 Cluj-Napoca, Romania; suciu_umf@yahoo.com; 4 CENCIRA Agrofood Research and Innovation Centre, Ion Meșter 6, 400650 Cluj-Napoca, Romania; daria.rus24@outlook.com; 5 The State Laboratory, Backweston Laboratory Campus, W23VW2C Celbridge, Ireland; mohammad.hossain@statelab.ie; 6 Department of Agriculture, Food and Animal Health, School of Health and Science, Dundalk Institute of Technology, Dublin Road, A91K584 Dundalk, Ireland

**Keywords:** grape pomace, secondary metabolites, mechanistic insights, sustainability, health, gut microbiota, functional foods, polyphenols

## Abstract

The growing interest in plant-derived bioactive secondary metabolites has renewed attention to grape pomace as a promising source within the context of sustainable and circular bioeconomy strategies. Its chemically diverse composition, influenced by cultivar, climate, and processing conditions, has shown a wide range of biological activities, including antioxidative, anti-inflammatory, antimicrobial, antidiabetic, and anticancer effects. Several reviews have addressed its composition, bioactivity, extraction, and applications, but a more integrated understanding of the molecular mechanisms is still needed. This review synthesizes recent evidence on the mechanistic actions of grape pomace metabolites, highlighting their involvement in key pathways such as Nrf2-mediated antioxidant defense, NF-κB-regulated inflammation, AMPK/SIRT1-dependent metabolic regulation, apoptosis-related signaling, and microbiota-driven phenolic metabolism. It also discusses challenges related to raw material variability, process standardization, and industrial scalability, and explores how advances in chemometrics, omics technologies, and data-driven optimization can support future development. Therefore, it provides an integrated perspective linking mechanistic insights with technological considerations to advance the sustainable valorization of grape pomace.

## 1. Introduction

### Secondary Metabolites of Grape Pomace and Their Relevance to Health

Plants synthesize a vast diversity of secondary metabolites—compounds not directly involved in primary metabolic processes such as growth or reproduction, but essential for ecological interactions and environmental resilience. These metabolites, which include phenolic compounds, flavonoids, tannins, stilbenes, terpenoids, alkaloids, and phytoalexins, contribute to plant defense against pathogens, herbivores, and abiotic stressors, and they facilitate communication and pollinator attraction [1].

In recent decades, secondary metabolites have attracted considerable attention for their broad spectrum of biological activities. Phenolic compounds have been linked to antioxidant, anti-inflammatory, antimicrobial, antidiabetic, cardioprotective, and anticancer effects [2,3]. More than 50,000 plant secondary metabolites have been identified, illustrating their immense chemical diversity and functional potential [4]. Increasing global interest in natural health-promoting compounds has further expanded the demand for plant-based secondary metabolites in functional foods, nutraceuticals, and pharmaceutical applications. Grape pomace (GP) has been shown to contain significant quantities of phenolics and flavonoids, often exceeding levels found in fresh produce [5,6].

In the wine industry specifically, GP represents a significant global by-product. Fruit pomace sources such as grapes and olives are consistently reported as the largest pomace volumes when compared with other fruits like apples, citrus, tomatoes, and peaches. The diversity of metabolites in GP makes it a valuable “reservoir” of bioactive and functional molecules. GP, which is the solid residue of skins, seeds, stems, and residual pulp remaining after winemaking, constitutes approximately 10–30% of the original grape mass [7]. With global grape production reaching 77.1 million tons in 2022 [8], the resulting pomace represents a substantial biomass resource. Typically, the production of 6 L of wine yields approximately 1 kg of GP, highlighting the magnitude of this underutilized byproduct. The composition of GP has been estimated at roughly 425 kg skins, 225 kg seeds, and 249 kg stalks per ton [7]. Each component contributes distinct phytochemicals: skins are rich in anthocyanins, flavanols, and polymeric proanthocyanidins, seeds contain high concentrations of catechins, epicatechins, gallic acid derivatives, and oligomeric proanthocyanidins, and stems, although less studied, contain tannins and phenolic acids with antimicrobial and antioxidant potential.

The chemical diversity of GP secondary metabolites is influenced by grape variety, geographical origin, vineyard practices, climatic factors, and winemaking conditions [9]. Despite this biochemical richness, large amounts of GP are still discarded or directed to low-value uses such as composting, representing a missed opportunity for value-added applications. At the same time, its composition is highly variable. The identity and abundance of GP metabolites depend strongly on grape variety, winemaking practices such as maceration and pressing, the inclusion of stalks or seeds, and post-processing conditions such as drying and extraction method [10].

**Figure 1:**
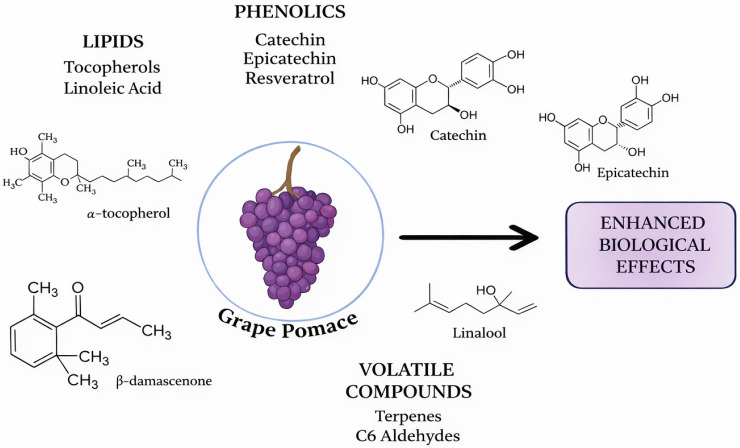
Different classes of metabolites in GP such as lipids, phenolics, and volatile compounds, interact to produce enhanced biological effects.

GP contains several metabolite classes, including phenolics, volatiles, lipids, and dietary fiber, which may interact within the matrix. These interactions can influence both biological properties and sensory characteristics (Figure 1) [10,11,12,13,14,15]. Phenolic compounds such as catechin, epicatechin, quercetin, resveratrol, and anthocyanins have been associated with antioxidant, anti-inflammatory, cardioprotective, and potentially metabolic effects. Volatile compounds, including alcohols, esters, terpenes, and aldehydes, contribute mainly to aroma and flavor and are therefore relevant to organoleptic quality. The lipid fraction, rich in unsaturated fatty acids and sterols, also has functional importance, both as a nutritional component and as a matrix that may affect the stability and delivery of lipophilic bioactive compounds such as tocopherols and some phenolics.

These combined properties support several potential applications of GP. Phenolic-rich fractions are relevant to functional foods and nutraceuticals, whereas volatile compounds may support uses in flavor, fragrance, or cosmetic formulations. In addition, the association of phenolics with dietary fiber has led to the concept of “antioxidant dietary fibre”, in which fiber-bound phenolics may contribute both antioxidant activity and gut-related benefits. Together, these features highlight GP as a multifunctional raw material with potential in food, nutraceutical, and cosmetic systems [10,11,12,13,14,15].

Although previous reviews have summarized GP composition, bioactivity, and applications, they have generally approached the topic from perspectives such as food applications, sustainable extraction, phenolic metabolism, therapeutic potential, and functional food development, while primarily providing descriptive overviews without integrating mechanistic evidence across experimental models or addressing translational relevance (Table 1). This review addresses that gap by examining how specific GP metabolites modulate defined molecular pathways, including Nrf2, NF-κB, AMPK/SIRT1, and apoptosis, and how they interact with the gut microbiota to influence systemic health. In addition, it discusses how raw material variability, processing methods, and formulation strategies affect bioavailability and functional outcomes. By linking chemical composition, mechanistic insight, and translational relevance, this review offers a more comprehensive and integrative perspective that can support the development of functional foods, nutraceuticals, and sustainable valorization strategies.

## 2. Mechanisms of Action and Biological Effects of Grape Pomace Bioactives

Understanding the health-related effects of GP requires consideration of the molecular mechanisms through which its secondary metabolites may exert biological activity (Figure 1). In vitro studies have consistently reported antioxidant, anti-inflammatory, and anticancer effects of GP-derived extracts and metabolites, whereas fewer studies in animal models have investigated the underlying cellular signaling pathways and target-specific interactions responsible for these responses. Human evidence remains comparatively limited, particularly with respect to mechanistic endpoints (Table 2).

### 2.1. Antioxidant Defense Modulation by Grape Pomace Polyphenols

Polyphenolic constituents of GP, including quercetin, catechins, and resveratrol, contribute to antioxidant protection primarily through modulation of endogenous cellular defense systems rather than acting solely as direct radical scavengers. Evidence from in vitro experiments and animal studies indicates that these compounds can enhance the expression and activity of cytoprotective and detoxifying enzymes—such as heme oxygenase-1 (HO-1), glutathione-related enzymes, and superoxide dismutase (SOD)—thereby improving cellular capacity to counteract oxidative stress and redox imbalance [16,17]. This indirect regulatory mechanism is increasingly considered more physiologically relevant than the transient antioxidant effects observed in chemical assays.

However, most mechanistic evidence derives from controlled cell-based systems and experimental animal models, often using concentrations or exposure conditions that may not reflect levels achievable in human tissues following dietary intake. In vivo responses are further influenced by absorption, metabolic transformation, and microbiota-mediated bioconversion of GP phenolics, which can alter both the identity and activity of circulating metabolites. Limited human evidence suggests that regular consumption of polyphenol-rich foods may modulate systemic antioxidant status, although the specific contribution of GP-derived compounds remains difficult to isolate. Overall, current evidence supports the view that GP polyphenols may enhance antioxidant defense through modulation of endogenous protective pathways, but this interpretation is based predominantly on preclinical evidence and requires further confirmation in well-designed human studies.

### 2.2. Regulation of Metabolic Homeostasis

In addition to redox modulation, GP-derived secondary metabolites have been associated with the maintenance of metabolic homeostasis, particularly under conditions of metabolic stress. Compounds such as resveratrol, catechins, and procyanidins influence multiple cellular processes involved in energy sensing, mitochondrial function, and substrate utilization. Specifically, these metabolites can activate signaling pathways that enhance mitochondrial biogenesis, increase fatty acid oxidation, regulate glucose uptake, and modulate insulin signaling, collectively supporting improved energy balance and lipid metabolism [18,19].

Evidence from preclinical models indicates that dietary supplementation with GP extracts can improve systemic markers of metabolic health, including glucose tolerance, serum lipid profiles, and overall energy homeostasis. Mechanistic studies suggest that these effects are mediated through coordinated modulation of metabolic signaling pathways, such as the AMPK–SIRT1 axis, which integrates nutrient and energy sensing with mitochondrial function. Furthermore, interactions with gut microbiota and the generation of bioactive metabolites may enhance or modify the systemic effects observed in vivo. Although human studies remain limited, preliminary data suggest that consumption of GP–enriched foods or extracts may support postprandial glycemic control and lipid metabolism, highlighting the translational potential of these bioactive compounds in functional foods and nutraceutical applications. Taken together, GP metabolites appear to exert multi-level, integrative effects on metabolic regulation, combining direct cellular pathway modulation with systemic and microbiota-mediated mechanisms, reinforcing their potential as bioactive ingredients for metabolic health [18,19].

### 2.3. Anticancer Effects of Grape Pomace-Derived Flavonoids and Proanthocyanidins

GP-derived flavonoids and proanthocyanidins exhibit multifaceted anticancer activities through diverse molecular mechanisms. These compounds can induce apoptosis in cancer cells via intrinsic mitochondrial pathways, including upregulation of pro-apoptotic proteins such as Bax, activation of caspase-9 and caspase-3, and downregulation of anti-apoptotic factors like Bcl-2. In parallel, they can promote cell cycle arrest at the G1 or G2/M phases, effectively halting uncontrolled proliferation. Beyond these classical cytotoxic effects, GP polyphenols also modulate key oncogenic signaling pathways, including PI3K/Akt, MAPK, and NF-κB, contributing to reduced survival signaling and impaired metastatic potential. Anti-angiogenic effects have been observed, characterized by inhibition of VEGF expression and disruption of endothelial cell proliferation, which may limit tumor vascularization and growth. Evidence for these effects comes from multiple cancer cell lines, including colon, breast, and prostate [20,21]. The matrix in which polyphenols are delivered, such as lipid-based carriers or whole GP extracts, may enhance cellular uptake, stability, and bioefficacy compared with isolated compounds in experimental systems. These observations provide a rationale for further translational investigation, including animal tumor models and, ultimately, human clinical studies. However, most available evidence at present derives from in vitro studies, and additional work is needed to determine whether these formulation-related effects remain relevant under physiological conditions and realistic dietary exposures.

### 2.4. Interaction of Grape Pomace Polyphenols with the Gut Microbiota

The gut microbiota plays a central role in modulating the health effects of GP polyphenols, and this interaction is bidirectional. On one hand, GP polyphenols and their breakdown products act as prebiotic-like agents, selectively stimulating beneficial bacteria such as *Bifidobacterium*, *Lactobacillus*, and *Akkermansia muciniphila*, while suppressing opportunistic or pathogenic species including *Clostridium perfringens* and *Escherichia coli* [22,23,24,25,26,27,28,29,30,31,32]. These microbiota shifts can enhance gut barrier integrity, reduce intestinal inflammation, and increase the production of short-chain fatty acids (SCFAs) such as acetate, propionate, and butyrate, which support energy homeostasis, immune signaling, and systemic metabolic health [33,34,35].

On the other hand, the gut microbiota metabolizes poorly absorbed GP polyphenols in the colon into bioactive metabolites, including urolithins, phenyl-γ-valerolactones, and other phenolic derivatives [22,23,24,25]. These microbial metabolites often exhibit enhanced bioactivity compared to the native compounds, influencing host antioxidant defenses, inflammatory pathways, and metabolic regulation. The bidirectional interaction between GP polyphenols and gut microbiota highlights that the systemic efficacy of GP depends not only on the native compounds but also on the metabolites generated by microbial biotransformation.

Together, these mechanisms indicate that GP polyphenols contribute to gut and systemic health through both direct modulation of microbial composition and indirect effects mediated by microbial-derived metabolites. Understanding this interplay is essential for designing functional foods or nutraceuticals that optimize the bioavailability and physiological impact of GP bioactives [22,23,24,25,26,27,28,29,30,31,32,33,34,35].

### 2.5. Microbial Metabolism and Bioactivity of Grape Pomace Polyphenols

Most grape polyphenols, particularly high molecular weight compounds such as proanthocyanidins, are poorly absorbed in the small intestine and therefore reach the colon largely intact. In the colon, these compounds undergo extensive microbial metabolism by the resident gut microbiota. Enzymatic activities of the microbiota convert complex polyphenols into smaller, low molecular weight metabolites, including phenylacetic acid, phenylpropionic acid, benzoic acid derivatives, and urolithins (in the presence of ellagitannins). These microbial metabolites often exhibit enhanced bioavailability compared with their parent compounds and can exert diverse systemic biological effects. Evidence suggests that these metabolites retain or even amplify key bioactivities of the original polyphenols, including antioxidant, anti-inflammatory, and anticancer effects. The specific metabolite profile is influenced by the host’s gut microbiota composition, the food matrix, and polyphenol structure, which contributes to inter-individual variability in physiological responses [26,27,28]. Understanding this microbiota-mediated metabolism is critical for interpreting in vivo efficacy, designing functional foods, and developing nutraceutical formulations, as the health benefits of GP polyphenols largely depend on the metabolites that ultimately circulate systemically.

### 2.6. Gut-Mediated Systemic Effects

The bioactive metabolites and the shifts in microbiota composition can lead to systemic effects, such as: reduced endotoxemia and systemic inflammation, improved insulin sensitivity, neuroprotective effects via the gut–brain axis, potential protective effects against colorectal cancer [36,37,38,39,40,41].

These outcomes suggest that the health effects of GP are partially mediated through the gut microbiome, positioning it as a functional dietary component that exerts postbiotic benefits [39,40,41].

**Figure 2:**
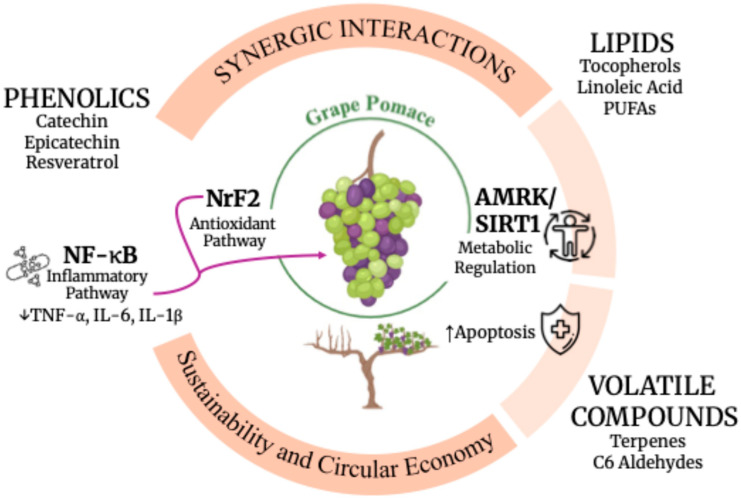
Grape pomace metabolic pathways (**↓**—decreased level, ↑—increased level).

GP is not only rich in individual classes of secondary metabolites—such as lipids, phenolics, and volatile terpenes—but also represents a complex biochemical matrix where these compounds interact to modulate biological activity (Table 3). Increasing evidence shows that the biological effects attributed to GP cannot be explained solely by isolated constituents [42]. Instead, synergistic interactions among metabolite classes enhance antioxidant capacity, anti-inflammatory signaling, antimicrobial activity, and gut microbiota modulation. These interactions are critical for understanding the whole-matrix bioactivity of GP and for optimizing extraction strategies that preserve multi-compound systems (Figure 2) [43].

### 2.7. Lipid–Phenolic Interactions Enhance Antioxidant and Anti-Inflammatory Activity

Grape seed oil within the pomace matrix typically contains 60–70% linoleic acid, 15–20% oleic acid, and minor lipophilic antioxidants including tocopherols (50–70 mg/100 g) and phytosterols [44]. These lipids interact strongly with polyphenols such as catechin, epicatechin, gallic acid, and proanthocyanidins [45].

### 2.8. Enhanced Bioavailability Through Lipid-Mediated Transport

Polyunsaturated fatty acids (PUFAs) increase membrane fluidity and facilitate the absorption of hydrophilic phenolics. The presence of grape seed oil has been shown to increase the intestinal uptake of catechins by 20–35%, improving intracellular antioxidant activity [46,47]. This results in enhanced activation of the Nrf2–Keap1 pathway, promoting the transcription of antioxidant enzymes (HO-1, NQO1, SOD).

### 2.9. Lipid–Phenolic Antioxidant Cycling

Tocopherols regenerate oxidized phenolics, prolonging their antioxidant half-life. Resveratrol and procyanidins, in turn, prevent PUFA peroxidation. This mutual stabilization contributes to sustained ROS neutralization and reduces lipid peroxidation markers such as MDA [48,49,50].

### 2.10. Suppression of NF-κB Inflammatory Signaling

Several in vitro and in vivo studies have shown that extracts derived from GP can reduce inflammatory responses in cellular models, often accompanied by decreased activation of the NF-κB signaling pathway and reduced expression of pro-inflammatory mediators such as TNF-α and IL-6 [51,52,53,54]. Procyanidin-rich fractions have similarly been associated with reduced inflammatory gene expression and cytokine secretion in macrophage and epithelial cell models, effects that have been linked to modulation of NF-κB signaling [55,56].

In addition to the intrinsic bioactivity of phenolic compounds, the surrounding matrix may influence their cellular availability and biological effects. Some studies suggest that lipid-containing systems can enhance the stability and cellular uptake of polyphenols, potentially leading to stronger anti-inflammatory responses in experimental models. For example, resveratrol and catechin delivered in lipid-based formulations, such as linoleic acid-derived matrices or nano-emulsions, have shown enhanced reductions in inflammatory markers compared with free compounds in certain cell-based studies [56,57]. While these observations suggest that matrix composition may influence phenolic bioactivity, the precise mechanisms underlying these effects and their relevance in vivo remain to be fully clarified. Overall, evidence indicates that GP metabolites may contribute to modulation of inflammatory responses, with NF-κB signaling representing one of several pathways potentially involved.

### 2.11. Activation of Nrf2 Antioxidant Responses

Polyphenols commonly found in GP—including catechins, procyanidins, anthocyanins, and resveratrol—have been reported in numerous studies to influence cellular antioxidant defense systems. In experimental models, these compounds have been associated with increased activity or expression of antioxidant enzymes such as heme oxygenase-1 (HO-1), NAD(P)H, quinone oxidoreductase-1 (NQO1), glutathione peroxidase (GPx), and superoxide dismutase (SOD), processes often linked to activation of the Nrf2 signaling pathway [58,59,60]. Extracts derived from GP have demonstrated the ability to reduce intracellular reactive oxygen species (ROS) and markers of lipid peroxidation, including malondialdehyde (MDA), in cellular models exposed to oxidative stress.

Procyanidin-rich fractions in particular have been associated with enhanced antioxidant responses and increased glutathione levels in several experimental systems [61]. It has been proposed that interactions between hydrophilic phenolics and lipophilic components naturally present in GP, such as fatty acids and tocopherols, may influence compound stability or membrane permeability, potentially affecting biological activity [56,62]. However, direct causal links between these matrix interactions and Nrf2 activation remain limited. Resveratrol has also been reported to influence upstream regulatory pathways, including AMPK and SIRT1, which in some experimental contexts have been associated with enhanced antioxidant responses and modulation of Nrf2 signaling [63]. Therefore, current evidence suggests that GP metabolites may contribute to cellular antioxidant defense, although the relative contribution of specific pathways and compound interactions requires further investigation.

### 2.12. AMPK/SIRT1-Related Metabolic Regulation and Apoptotic Responses

Beyond their antioxidant and anti-inflammatory properties, GP polyphenols have been investigated for their potential effects on cellular metabolism and apoptosis in experimental models. Compounds such as resveratrol, catechins, and procyanidins have been reported to influence cellular energy-sensing pathways, including the AMPK–SIRT1 axis, which plays a key role in metabolic regulation and mitochondrial function [63,64,65]. In several in vitro and animal studies, activation of AMPK has been associated with increased mitochondrial biogenesis, enhanced fatty acid oxidation, and modulation of metabolic regulators such as PGC-1α. These processes may contribute to improved cellular resilience under conditions of metabolic or oxidative stress.

GP phenolics have also been investigated for their potential pro-apoptotic effects primarily in cancer cell models. In vitro studies have reported mechanisms including alterations in mitochondrial membrane potential, changes in the Bax/Bcl-2 ratio, release of cytochrome-c, and activation of downstream caspases such as caspase-9 and caspase-3 [66,67,68,69]. Procyanidins have been associated with modulation of signaling pathways including MAPK and PI3K/Akt in specific experimental systems [70]. Similarly, resveratrol has been reported to interact with multiple signaling pathways, including AMPK and p53, under certain experimental conditions [71]. Overall, these findings indicate plausible mechanistic routes through which GP metabolites may influence apoptosis, but the current evidence is derived mainly from controlled in vitro studies, with limited confirmation in animal models and insufficient human evidence to establish their physiological relevance.

## 3. Biological Activities of Grape Pomace-Derived Metabolites

Overall, GP yields complex mixtures of bioactive metabolites, notably polyphenolic compounds (anthocyanins, flavan-3-ols, phenolic acids, stilbenes), and non-phenolic molecules such as triterpenoids. These fractions show antioxidant, redox-modulating, anti-inflammatory, antimicrobial, antidiabetic, and anticancer properties chemical, cellular, and animal models. Key bioactivities are strongly influenced by structural attributes (e.g., degree of hydroxylation, polymerization) and by biotransformation processes that can enhance metabolite accessibility and potency [72,73,74,75]. 

### 3.1. Antioxidant and Redox-Modulating Properties

Evidence from chemical, in vitro, and in vivo studies demonstrates that GP extracts and derived metabolites can scavenge free radicals (DPPH, ABTS+, hydroxyl radicals), inhibit lipid peroxidation, ROS, and enhance endogenous antioxidant defenses such as catalase (CAT), SOD, and GPx activities in biological systems. These effects are generally linked to total phenolic content and the specific structural features of constituent metabolites such as hydroxylation patterns and conjugated systems that facilitate radical stabilization and electron donation. For example, fermentation of GP can increase the availability of simpler phenolics like catechin, epicatechin, and procyanidins, which exhibit strong antioxidant and antiproliferative actions in colorectal cancer models (Caco-2, SW620) through oxidative stress modulation and induction of apoptosis pathways [76,77].

The antioxidant capacity of GP polyphenols is modulated by structural elements such as the number and position of hydroxyl groups, degree of polymerization (e.g., procyanidin oligomers), and conjugation. Highly hydroxylated flavonoids and polymeric proanthocyanidins tend to exhibit higher radical scavenging and ferric reducing activities than simpler phenolic acids due to enhanced electron donation and stabilization of phenoxyl radicals.

### 3.2. Anti-Inflammatory Effects

GP-derived polyphenols exert anti-inflammatory actions in both in vitro and in vivo settings by modulating pro-inflammatory signaling pathways and cytokine release. Specific GP fractions have demonstrated the capacity to attenuate inflammatory responses in vivo, particularly in experimental models of acute inflammation. These effects are reflected in reduced tissue oxidative damage, leukocyte infiltration, and inflammatory enzyme activity, indicating a broad immunomodulatory action rather than isolated cytokine suppression. Therefore, these findings support the role of GP-derived metabolites in limiting inflammation-associated oxidative stress and preserving tissue integrity. GP polyphenolic fractions attenuate carrageenan-induced paw edema and the expression of key inflammatory enzymes and cytokines in dose-dependent manners, suggesting potential utility as natural anti-inflammatory agents [78].

### 3.3. Antimicrobial and Antidiabetic Activities

GP extracts also show antimicrobial activity, particularly against pathogenic bacteria such as *Escherichia coli*, *Listeria monocytogenes*, and *Bacillus megaterium*, driven by phenolic compounds including anthocyanins, flavonoids, and phenolic acids. In vitro simulated gastrointestinal digestion of GP can enhance extract accessibility and influence antimicrobial potency, likely via release of bioactive metabolites during digestion [79].

Regarding antidiabetic activity, GP-derived metabolites inhibit key carbohydrate-digesting enzymes such as α-glucosidase, resulting in suppressed postprandial hyperglycemia in animal models of diabetes. In addition, individual triterpenoids such as oleanolic acid, identified in red GP, have been proven as promising antidiabetic molecules through modulation of glucose uptake and mitochondrial activity in muscle cell models [80,81].

### 3.4. Anticancer and Antimutagenic Properties

GP phenolic extracts have demonstrated anticancer and chemo-preventive potential in multiple in vitro cancer models. Phenolic-rich GP extracts can inhibit proliferation, induce apoptosis, and cause cell cycle arrest in cancer cell lines such as Caco-2 and SW620 (colorectal carcinoma), and reductions in tumor cell growth have been observed with fermented GP preparations. These effects are often attributed to both antioxidant activity (reducing oxidative DNA damage) and pro-apoptotic signaling pathways activated by flavonoids and other phenolics. Furthermore, GP extracts exhibit antimutagenic properties in systems exposed to oxidative stressors, potentially reducing DNA damage induced by free radicals [77,82].

The existing evidence highlights the therapeutic potential of GP-derived metabolites as natural functional compounds, while there is an increasing need for further mechanistic and clinical studies to translate these findings into practical applications (Table 4).

## 4. Bioprocessing and Biotransformation Approaches

A wide range of physical, chemical, and thermal processing techniques have been applied to GP; however, enzymatic treatments and microbial fermentation are highlighted in this section because they constitute the most targeted, sustainable, and functionally impactful biotransformation strategies currently available [90]. Unlike conventional methods, such as solvent extraction, high-temperature processing, ultrasonication, or chemical hydrolysis, which primarily improve phenolic recovery without substantially altering molecular structures, enzymatic and fermentation-based approaches actively convert complex phenolics into more bioavailable and bioactive metabolites through selective biochemical reactions [91]. Enzymatic hydrolysis enables precise modification of polymeric tannins, cell wall polysaccharides, and glycosylated flavonoids, while fermentation introduces additional metabolic pathways capable of generating novel compounds and enhancing biological potency. Both strategies align with green bioprocessing principles, operate under mild conditions, and are scalable for industrial integration. Thus, the emphasis on these two bioprocessing routes reflects their demonstrated superiority in enhancing phenolic functionality, their relevance to circular bioeconomy models, and their strong support in contemporary scientific literature compared with other, less transformative processing methods (Table 5).

### 4.1. Enzymatic Treatments to Modify Phenolic Profiles

Enzymatic bioprocessing represents an effective strategy to modify and enhance the phenolic composition of GP, improving both extractability and bioactivity of its metabolites. Enzymes such as cellulases, hemicellulases, pectinases, tannases, and β-glucosidases are applied to disrupt the complex polysaccharide–polyphenol matrix of grape cell walls, promoting the release of bound phenolics and the transformation of high-molecular-weight compounds into smaller, bioavailable forms. Different enzymatic systems contribute to distinct transformation pathways. Cell wall-degrading enzymes such as cellulases, hemicellulases, and pectinases facilitate the release of anthocyanins, flavan-3-ols, and phenolic acids, producing extracts with improved antioxidant, anti-inflammatory, and antimicrobial activity in experimental systems. Enzymatic hydrolysis has also been associated with a reduction in the degree of polymerization of proanthocyanidins, enhancing radical scavenging effectiveness and cellular uptake. Tannase catalyzes the hydrolysis of tannins and galloylated catechins into simpler molecules such as gallic acid—metabolites associated with reported antioxidant, anti-inflammatory and antimutagenic potentials [87,96,97,98,99]. In addition, β-glucosidase hydrolyzes glycosidic bonds and releases aglycone forms from glycosylated substrates, transformation that may improve bioavailability because flavonoid aglycones generally show improved intestinal availability and metabolic stability compared to their corresponding glycosides. Overall, the enzymatic treatments not only enhance extraction yields but also strategically shape the phenolic profile toward greater biological efficacy and functional value.

### 4.2. Fermentation for Enhanced Bioactivity

**Figure 3:**
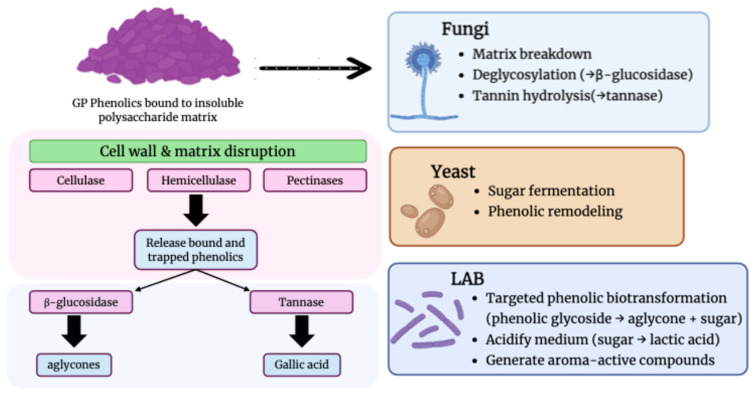
Fermentation-Driven Biotransformation of Bound Grape Pomace Phenolics.

Fermentation, either through solid-state fermentation (SSF) or submerged fermentation (SmF), represents a complementary bioprocessing strategy in which microbial metabolism promotes both the release and transformation of GP phenolics (Figure 3). In this context, filamentous fungi, lactic acid bacteria (LAB)**,** and yeasts play distinct but overlapping roles. Fungal fermentation is particularly effective in degrading the plant matrix because fungi produce abundant extracellular hydrolases that facilitate the release of soluble phenolics from insoluble cell-wall-associated forms. For example, SSF of GP seeds with *Aspergillus niger*, *Monascus anka*, and *Eurotium cristatum* increased the soluble phenolic fraction and improved antioxidant activity, with marked increases in compounds such as chlorogenic acid, syringic acid, ferulic acid, epicatechin gallate, and resveratrol [100]. In addition, *Aspergillus* species are relevant producers of tannase and β-glucosidase, making them especially important for de-galloylation and deglycosylation reactions during GP valorization [87,96,97]. LAB are more closely associated with hydrolytic fine-tuning of phenolic profiles during fermentation. Their β-glucosidase activity can convert glycosylated phenolics into aglycones, thereby increasing the proportion of compounds with potentially greater bioavailability and biological activity [96]. LAB fermentation has also been linked more broadly to improved functionality of GP-derived products, including reduced sugar content, altered phytochemical composition, and the formation of new derivative compounds that affect both aroma and health-related properties [23]. Yeasts, in turn, contribute primarily through the fermentation of residual sugars and metabolic interactions that may alter aroma-active precursors and favor the formation of lower-molecular-weight phenolic derivatives, although the exact mechanisms by which yeasts contribute are often discussed together with the broader technological benefits of fermentation [23].

## 5. Factors Affecting Bio Accessibility

The bio accessibility of GP metabolites, e.g., the fraction released from the food matrix during digestion and available for absorption, is governed by a complex interplay of physicochemical, biological, and processing-related factors. Understanding these factors is essential to translate in vitro antioxidant/biological activities into in vivo effects and to design processing or formulation strategies that improve functional outcomes.

### 5.1. Food Matrix and Physical Structure

GP is a heterogeneous material composed of skins, seeds, stems and a high proportion of dietary fiber (both insoluble and soluble). Phenolics in GP are frequently bound to cell-wall polysaccharides (cellulose, hemicellulose, pectin) or sequestered inside plant tissue micro-compartments, which limits their release in the stomach and small intestine and reduces early absorption. Mechanical and structural constraints—particle size, porosity and the thickness/integrity of cell walls—therefore strongly determine how much phenolic material becomes bio accessible during gastrointestinal transit. In vitro digestion studies of fruit and pomace matrices consistently show that smaller particle sizes and disrupted cell walls increase early release, while intact fiber matrices protect bound phenolics until colonic fermentation [98,99].

### 5.2. Molecular Structure of Phenolics

Intrinsic chemical features, such as molecular weight, degree of polymerization, glycosylation, hydroxylation pattern and esterification, are primary determinants of whether a given phenolic will be solubilized, absorbed in the small intestine, or pass to the colon. Low-molecular-weight aglycones and simple phenolic acids are generally more water-soluble and better substrates for small-intestinal uptake, whereas polymeric proanthocyanidins and ellagitannins are poorly absorbed intact and reach the microbiota where they are depolymerized into smaller, absorbable metabolites. Glycosides often require deglycosylation (by intestinal or microbial glycosidases) before absorption; methylation and galloylation reduce passive diffusion and can lower small-intestinal uptake. These structure–function relationships strongly condition the bio accessibility profile across GP phenolic subclasses [100,101].

### 5.3. Processing and Pre-Treatments (Mechanical, Thermal, Enzymatic, Fermentation)

Processing can either enhance or reduce bio accessibility depending on method and intensity. Mild, targeted treatments that disrupt the plant matrix (milling, controlled thermal treatments, high-pressure processing, enzymatic hydrolysis with pectinases/cellulases/β-glucosidases) typically increase the fraction of phenolics released during simulated gastric and intestinal digestion and can increase the pool available for absorption in the small intestine. Enzymatic and fermentation interventions additionally bio transform bound/polymeric phenolics into simpler compounds (aglycones, phenolic acids, low-DP flavan-3-ols) that are more bio accessible and sometimes more bioactive. The thermal or oxidative processing can degrade heat-sensitive anthocyanins and other labile compounds, decreasing bio accessibility. Systematic reviews and experimental work on GP and other phenolic-rich matrices report consistent gains in bio accessibility after enzymatic treatment and controlled fermentation, while high-temperature or prolonged oxidation tends to reduce recoverable, active phenolics [102].

### 5.4. Interactions with Macronutrients and Other Food Components

During digestion, polyphenols interact with dietary proteins, lipids, carbohydrates and minerals; these interactions can either sequester phenolics (reducing apparent bio accessibility) or enhance transfer into absorbable forms (e.g., micellar incorporation with lipids for lipophilic polyphenols). Protein–polyphenol complexes (including milk proteins) may reduce free polyphenol concentration but sometimes protect phenolics from degradation and facilitate intestinal uptake depending on the system. Dietary lipids generally promote absorption of more hydrophobic phenolics by aiding their incorporation into mixed micelles, whereas viscous carbohydrate matrices and high-fiber meals can entrap polyphenols and delay or reduce small-intestinal availability—often redirecting them to colonic metabolism. The effect is strongly context-dependent and varies with compound polarity and the physicochemical environment of the meal [103].

### 5.5. Role of the Gut Microbiota and Colonic Bio Accessibility

Because a large proportion of GP phenolics (especially high-DP tannins and glycosylated flavonoids) reach the colon, the microbiota governs a second, critical phase of bio accessibility by metabolizing complex phenolics into smaller, often more absorbable and sometimes more bioactive compounds (e.g., phenyl-γ-valerolactones, protocatechuic and hydroxy phenylacetic acids). The composition and enzymatic repertoire of an individual’s microbiota (presence of tannase producers, glycosidase activity, decarboxylases, reductases) therefore defines which metabolites appear in plasma and urine after pomace intake, and how long they persist systemically. In turn, polyphenols selectively modulate microbial populations, promoting taxa linked to health (e.g., *Lactobacillus*, *Bifidobacterium*, *Akkermansia*), establishing a bidirectional diet–microbiota relationship that shapes long-term bio accessibility and biological outcomes [104,105].

### 5.6. Host Physiology and Inter-Individual Variability

Host factors play a critical role in determining the bioaccessibility, absorption, metabolism, and ultimately the physiological effects of GP phenolics. Variables such as age, gastric emptying rate, intestinal transit time, bile secretion, and the expression of intestinal transporters and conjugating enzymes—including UDP-glucuronosyltransferases (UGTs), sulfotransferases (SULTs), and catechol-O-methyltransferase (COMT)—can substantially influence the fraction of phenolics that become systemically available and their subsequent metabolic fate. In addition, concomitant use of medications, such as proton pump inhibitors or antibiotics, can further modify gastrointestinal conditions, alter microbiota composition, and influence enzymatic activity, thereby impacting polyphenol absorption and biotransformation [103,106,107].

Genetic polymorphisms in phase II metabolizing enzymes contribute to variability in the conjugation and clearance of GP-derived phenolics, affecting circulating levels of glucuronides, sulfates, and methylated metabolites. Differences in gut microbiome composition also play a major role in shaping individual metabolic profiles, since microbial communities mediate the deglycosylation, ring-fission, and reduction of complex polyphenols, generating metabolites with altered bioactivity and bioavailability. Consequently, the same GP preparation can lead to highly variable circulating metabolite profiles and physiological responses across individuals, even when administered at identical doses.

Other host-specific factors—including body mass, sex, nutritional status, and habitual diet—may modulate the absorption and metabolism of GP phenolics by influencing gastrointestinal physiology, microbial ecology, and enzyme expression. For instance, high-fat or high-protein meals can alter bile acid secretion and micelle formation, thereby affecting the solubilization and uptake of lipophilic phenolic compounds. Age-related changes in intestinal barrier function, enzyme expression, and microbiome composition further contribute to inter-individual differences, particularly in pediatric and elderly populations.

This inter-individual variability has important implications for the design of human studies, dietary interventions, and functional food applications. Understanding host-related determinants of GP phenolic metabolism can inform strategies to enhance bioavailability, such as formulation in lipid-rich matrices, microencapsulation, co-administration with prebiotics, or personalized nutrition approaches. Ultimately, integrating knowledge of host genetics, physiology, and microbiota composition is essential for predicting the efficacy of GP-derived bioactives and for translating promising in vitro or animal model findings into consistent, physiologically meaningful outcomes in humans.

### 5.7. Experimental Approaches for Evaluating Bioaccessibility

The evaluation of GP’ phenolic bioaccessibility commonly relies on in vitro digestion models, which are used to estimate the fraction of compounds released from the food matrix during gastrointestinal transit and therefore potentially available for absorption. Among these, static in vitro digestion systems are the most widely used because they are relatively simple, reproducible, and suitable for comparative screening studies. In particular, the harmonized INFOGEST static digestion protocol has become an important reference method, as it standardizes experimental variables such as pH, enzyme activity, digestion time, electrolyte composition, and bile content across the oral, gastric, and intestinal phases. In the context of grape-derived phenolics, static in vitro digestion models have been widely applied to grapes, wine, and GP fractions to assess phenolic release, transformation, and recovery under simulated gastrointestinal conditions. Compared with static systems, dynamic digestion models attempt to reproduce gastrointestinal processes more realistically by incorporating changing pH conditions, gradual addition of digestive fluids, mixing, and gastric emptying. Although these systems are more physiologically relevant, they are also more technically demanding, more expensive, and less accessible for routine food research applications. To investigate the step between release and absorption, more advanced in vitro studies often combine digestion models with intestinal epithelial systems, especially Caco-2 cell monolayers, which are widely used to mimic the intestinal barrier. These models allow researchers to assess epithelial transport, paracellular permeability, and the influence of digested extracts or metabolites on barrier-related markers. As also noted in broader methodological overviews, Caco-2 models are commonly used in advanced in vitro studies to examine the intestinal transport of bile acids and phenolic compounds, although they still represent a simplified approximation of the in vivo intestinal environment [83,90].

Due to the fact that many GP phenolics, especially polymeric proanthocyanidins and glycosylated compounds, are only partially absorbed in the small intestine, colonic fermentation systems are also highly relevant for bioaccessibility studies. These models help characterize microbiota-mediated biotransformation of GP phenolics into smaller metabolites that may show greater absorbability or altered biological activity. They are especially relevant for GP because gut microbial metabolism is likely to play a major role in determining which metabolite forms ultimately reach systemic circulation.

The interpretation of bioaccessibility experiments also depends on the analytical techniques used to characterize native compounds and digestion-derived metabolites. More broadly, these approaches align with targeted and untargeted metabolite profiling strategies that can help distinguish between compounds that are merely extractable and those that remain stable, become bioaccessible, or are microbiota-derived. Taken together, these approaches provide a more realistic framework for assessing how GP-derived compounds are released, transformed, and made available for absorption.

### 5.8. Formulation Strategies to Improve Bio Accessibility

Practical strategies to increase GP phenolic bio accessibility include enzymatic pre-treatment and microbial fermentation (to release and convert bound phenolics), microencapsulation (to protect labile anthocyanins through gastric transit and enable targeted intestinal release), and co-formulation with lipids or emulsifiers for hydrophobic compounds. Encapsulation can improve stability and controlled release, but its success depends on carrier chemistry and release kinetics; some encapsulation systems can inadvertently reduce small-intestinal absorption if they release payloads primarily in the colon [108,109,110]. Integrative approaches that combine matrix disruption (milling/enzymes), targeted fermentation, and rational delivery systems are the most promising for converting GP into nutraceutical ingredients with predictable bio accessibility and activity.

Therefore, the bio accessibility of GP metabolites is not a single property of the compound but an emergent property of compound chemistry × food matrix × processing × gastrointestinal environment × microbiota × host physiology. For translational applications (functional foods, supplements, cosmeceuticals) it is therefore essential to pair compositional analyses with standardized in vitro digestion and colonic fermentation models, followed by targeted metabolite profiling in human trials to validate that processing gains in extractable phenolics translate into increased circulating metabolites and measurable biological effects.

## 6. Potential Applications and Dietary Intake

### 6.1. Functional-Food Ingredients and Dietary Supplements

GP is a nutrient and phytochemical-dense by-product. The compositional attributes make GP attractive as a multifunctional ingredient in the formulation of functional foods and dietary supplements [110,111,112,113]:

Bakery and cereal products—incorporation of GP flour or powder to increase dietary fiber and antioxidant capacity (improves nutritional profile; may alter color and sensory attributes that must be optimized).

Dairy and fermented foods—GP powders and extracts have been used to fortify yogurts, cheeses and beverages; fermentation with LAB can further generate bioactive aglycones and increase bio accessibility.

Meat products and meat analogues—polyphenol-rich GP fractions act as natural antioxidants and colorants, improving oxidative stability and reducing lipid oxidation in processed meats.

Nutraceutical extracts and standardized supplements—concentrated GP extracts (standardized for total polyphenols, proanthocyanidins, or resveratrol) are marketed as antioxidant and cardiometabolic supplements; clinical and exercise studies commonly use 100–1000 mg total polyphenols/day formulations.

Functionally, GP as ingredients, it can (i) increase dietary fiber and prebiotic substrate for the colon, (ii) supply antioxidant phenolics that modulate redox and inflammatory pathways (in vitro/in vivo evidence), and (iii) act as food-grade preservatives or colorants. However, practical application requires tailoring of particle size, sensory masking, extraction/standardization and stability (anthocyanins are labile to pH and heat), and demonstration that bioactive gains survive processing and digestion.

### 6.2. Inclusion in Animal Feed and Feed-Derived Nutraceuticals

GP has been widely investigated as an animal feed supplement for ruminants, monogastrics (pigs, poultry), and aquaculture species, offering both a low-cost source of dietary fiber and energy as well as a rich source of bioactive compounds with potential health and product-quality benefits [111,114,115,116]. Its inclusion in animal diets provides an opportunity to valorize a large-volume agri-food by-product while contributing to circular economy strategies.

Ruminants: GP is generally incorporated at moderate inclusion rates (typically up to 10–15% of dry matter, depending on composition and tannin content). In ruminants, the rumen microbiota can partially buffer the potential anti-nutritional effects of tannins, allowing higher inclusion levels without negatively affecting digestibility. Benefits observed include improved fatty acid profiles in meat and milk, with higher levels of unsaturated fatty acids and conjugated linoleic acid, as well as increased antioxidant content in derived products. GP supplementation has also been associated with modulation of rumen microbial populations, reduction in methane emissions, and improved animal health parameters such as oxidative stress markers and immune status.

Monogastrics (poultry, pigs): The higher crude protein and fiber content, as well as tannin levels, limit the maximum inclusion rate in monogastric diets, with typical supplementation ranging from 2–6% to avoid adverse effects on palatability, digestibility, and growth performance. Within these ranges, GP has been shown to enhance meat quality, including improved color, lipid stability, and antioxidant capacity. Some studies also report positive modulation of gut microbiota, supporting intestinal health and nutrient absorption. However, outcomes can be variable, depending on factors such as GP composition, animal age, and diet formulation.

Aquaculture species: Recent research has explored GP supplementation in fish diets, where polyphenols and fiber can influence gut microbiota, immune response, and oxidative status. Moderate inclusion rates have been linked to enhanced growth efficiency, improved fillet quality, and increased resistance to oxidative stress, although high tannin content may limit digestibility and feed intake.

Functional feed additives and microencapsulation: To overcome limitations associated with bulk GP inclusion and anti-nutritional factors, GP extracts or microencapsulated polyphenols have been trialed as functional feed additives. Microencapsulation allows the delivery of concentrated antioxidants in a controlled-release format, reducing negative effects on feed palatability while enhancing oxidative stability of animal products. Such preparations have been shown to improve feed conversion efficiency, modulate gut microbiota composition, and reduce lipid peroxidation in meat, milk, and eggs. Additionally, targeted delivery can enhance the bioavailability of polyphenols, enabling their systemic effects, including modulation of inflammation, antioxidant defense, and metabolic pathways.

Processing and formulation considerations: The successful inclusion of GP in animal feed depends on careful matching to species-specific physiology and digestive characteristics. For example, ruminants can tolerate higher fiber and tannin levels than monogastrics. Stabilization methods such as drying, ensiling, or pelleting can influence metabolite preservation, palatability, and shelf-life. Tannin content, fiber composition, and moisture level must be monitored to optimize inclusion rates and ensure consistent product quality. In addition, GP can be blended with other feed components or supplemented with enzymes to improve digestibility and nutrient availability.

Incorporating GP into animal diets provides an environmentally and economically sustainable valorization route, simultaneously reducing agro-industrial waste, lowering feed costs, and enhancing the nutritional and functional quality of animal-derived products. When combined with emerging feed technologies—such as encapsulation, fermentation, and tailored diet formulations—GP represents a flexible strategy to integrate by-products into precision livestock and aquaculture systems, aligning with circular economy and sustainable nutrition objectives. Future research should focus on optimizing processing and inclusion strategies to balance bioactivity with digestibility, standardizing GP composition through quality control measures, and evaluating long-term effects on animal health, productivity, and product quality. Additionally, understanding species-specific metabolic handling of GP polyphenols and fibers will be key to designing functional feeds that deliver both health benefits and sustainable valorization outcomes.

### 6.3. Safety and Toxicity Concerns

Although GP contains many potentially beneficial molecules, safety must be explicitly addressed before large-scale human consumption or high inclusion in animal diets. Based on risk magnitude and potential impact, key safety concerns can be prioritized as follows [12,117,118,119,120,121]:

(a) Contaminants (Highest risk)

GP can accumulate heavy metals (e.g., Cu, Zn, Pb, Cd, Mn, Ni, As) and pesticide residues from vineyard practices. Depending on geographical and agronomic factors, concentrations may approach regulatory limits. Routine screening is therefore essential for GP intended for food or feed applications. Mitigation strategies include supply-chain control, washing, detoxification (e.g., activated carbon), and sourcing from low-contaminant vineyards [12,117,118,119,120,121].

(b) Mycotoxins and microbial hazards

Poorly handled or stored pomace may harbor molds producing mycotoxins such as ochratoxin A, aflatoxins, or zearalenone. While GP has been investigated as a biosorbent to reduce mycotoxin absorption in vivo, the presence of mycotoxins in raw material requires careful monitoring and mitigation. Good agricultural practices, rapid drying or ensiling, and detoxification processes are recommended.

(c) Anti-nutritional factors and tannins

High levels of condensed tannins and other phenolics can reduce protein digestibility and mineral bioavailability by forming complexes. Inclusion rates must therefore be optimized for specific species in feed, or processed (e.g., enzymatic hydrolysis, fermentation) to reduce polymeric tannins and enhance bioactive aglycone availability.

(d) Drug–nutrient interactions and systemic effects

Concentrated GP polyphenols may interact with drug-metabolizing enzymes (phase I/II), transporters, or platelet function, and certain molecules may exhibit estrogenic activity. Standard toxicology assessment and knowledge of circulating bioactive metabolites are necessary to support supplement claims and regulatory compliance.

(e) Allergenicity and sensory acceptability (Lowest risk)

GP contains residual proteins that could, in principle, act as allergens. While no widespread allergenicity has been reported, any new food ingredient requires formal allergen risk assessment and consideration of sensory impacts, such as color, astringency, or off-flavors.

As risk-management recommendations it can be considered a routine screening for heavy metals/pesticides/mycotoxins; specification of acceptable inclusion ranges (species-specific for feed); processing steps (drying, ensiling, enzymatic/fermentation detoxification); standardization of extracts; and clinical toxicology data for concentrated nutraceuticals. Table 6 summarizes the ranked safety risks of GP and the corresponding regulatory considerations for whole powders vs. standardized extracts.

### 6.4. Estimated Daily Intake (EDI) Through Functional Foods

Estimating the likely daily intake of GP-derived polyphenols from formulated foods/supplements requires data on the TPC of the GP material, the inclusion level in the food, and the serving size (Table 7). TPC of GP varies widely by grape variety, winemaking technique and extraction method (reported ranges in the literature typically span ~10–200 mg GAE/g dry pomace, with many values clustering 20–80 mg GAE/g for whole-pomace powders and higher values for concentrated extracts) [42].

## 7. Gaps in Research and Future Directions

**Figure 4:**
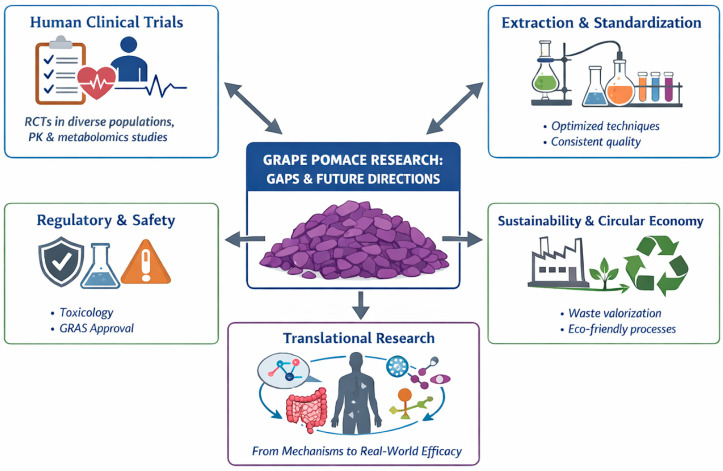
Grape pomace critical gaps. This schematic summarizes the main unresolved challenges that limit the translational application of GP-derived metabolites.

Despite significant advances in GP valorization, several critical gaps remain that must be addressed to support clinical translation, industrial application, regulatory compliance, and sustainability of GP-derived metabolites (Figure 4).

### 7.1. Need for Clinical Trials and Human Evidence

Although in vitro and preclinical in vivo studies consistently demonstrate the antioxidant, anti-inflammatory, antidiabetic, and anticancer potential of GP-derived polyphenols and their metabolites, well-designed human clinical evidence remains limited. To date, many of the reported health benefits associated with GP are derived from observational studies or small-scale pilot interventions, rather than from randomized controlled trials (RCTs) capable of establishing robust cause–effect relationships. In the absence of clinical validation of safety, efficacy, and effective intake levels, the translation of GP bioactive compounds into functional foods and nutraceutical formulations remains hypothetical. A further limitation is the lack of harmonized clinical endpoints relevant to secondary metabolite activity. Biomarkers related to oxidative stress, inflammatory status, glucose and lipid metabolism, and gut microbiome modulation are not consistently assessed or standardized across human studies, challenging evidence integration and comparison and evidence synthesis [117]. In addition, interindividual variability in absorption, metabolic transformation, gut microbiota composition, genetic background, and habitual diet significantly influences biological responses to GP intake, highlights the need for adequately informed human studies.

Future research should therefore prioritize randomized, placebo-controlled trials designed to evaluate dose–response relationships, long-term safety, and metabolic outcomes of GP extracts and GP-enriched foods in diverse populations, including individuals at increased risk of metabolic disorders, cardiovascular disease, or gut dysbiosis. Importantly, the integration of pharmacokinetic assessments with targeted and untargeted metabolomics will be essential to identify which GP-derived metabolites reach systemic circulation following realistic dietary exposure or supplementation. Such approaches will strengthen the biological plausibility of observed effects and provide a solid foundation for evidence-based health claims [118].

### 7.2. Improved Extraction, Standardization, and Characterization

A major limitation in current GP research is the considerable heterogeneity in extraction methods, analytical protocols, and bioactivity assays used across different studies. This methodological variability complicates direct comparison of results and limits the robustness of conclusions regarding the composition and biological activity of GP-derived metabolites. GP represents a chemically complex matrix containing multiple classes of compounds—including polyphenols, dietary fibers, polysaccharides, proteins, and residual lipids—whose extraction efficiency depends strongly on processing conditions. Differences in solvent composition, extraction time, temperature, particle size, and pre-treatment strategies can substantially influence both the qualitative and quantitative profiles of recovered metabolites [119]. The reported concentrations of phenolic compounds and antioxidant activities may vary widely across studies, even when similar grape cultivars or processing residues are examined. In addition to extraction variability, analytical approaches used to characterize GP extracts also differ significantly among studies. Many investigations rely on global indicators such as TPC and general antioxidant assays (e.g., DPPH, ABTS, or FRAP). While these assays are useful for preliminary screening, they do not adequately capture the structural diversity and polymerization degree of phenolic compounds, which can strongly influence their biological activity [120]. Moreover, differences in assay conditions, calibration standards, extraction yields, and reporting units further reduce the comparability of results between independent studies. As a consequence, linking specific extraction conditions to particular phenolic profiles or bioactivities remains challenging.

Bioactivity evaluation represents another important source of heterogeneity. Studies investigating GP-derived extracts frequently employ different experimental systems, including diverse cell lines, animal models, or biochemical assays, often using varying extract concentrations, exposure times, and endpoints. These methodological differences may lead to inconsistent findings and complicate attempts to establish clear relationships between extract composition and biological effects. In some cases, bioactivity outcomes are reported without detailed chemical characterization of the tested extracts, which further limits the ability to identify the compounds responsible for observed biological responses. Conventional extraction approaches for GP typically rely on organic solvents such as methanol, ethanol, or acetone, which are effective for recovering phenolic compounds but may raise concerns related to environmental sustainability and solvent residues when extracts are intended for food or nutraceutical applications [121]. In response to these limitations, several emerging “green” extraction technologies have been proposed, including supercritical fluid extraction (SFE), subcritical water extraction, enzyme-assisted extraction, and natural deep eutectic solvents (NADES). These approaches may improve selectivity and reduce solvent consumption; however, their application remains largely limited to laboratory-scale investigations, and their performance under industrial conditions has not yet been fully validated [122,123]. Furthermore, the influence of these extraction strategies on metabolite stability, bioaccessibility, and biological activity remains insufficiently characterized. Given these challenges, improved standardization of extraction procedures and analytical characterization is essential to enhance the reliability and comparability of GP research. Reliance on single metrics such as TPC alone is insufficient to describe the chemical complexity of GP extracts. Instead, multi-parameter characterization approaches—including detailed profiling of proanthocyanidins, anthocyanins, and other phenolic subclasses, as well as quantification of representative marker compounds and assessment of polymerization degree, should be more widely adopted. Advanced analytical tools such as high-resolution mass spectrometry, metabolomics, and chromatographic fingerprinting can provide comprehensive compositional information and facilitate batch-to-batch consistency assessment.

Overall, addressing the current heterogeneity in extraction techniques, analytical protocols, and bioactivity assays is critical for improving the reproducibility and interpretability of GP research. Greater methodological transparency, standardized reporting of experimental conditions, and comprehensive chemical characterization will strengthen the evidence base required to link GP metabolite composition with biological activity and support the development of scientifically substantiated food and nutraceutical applications.

Furthermore, the lack of inter-laboratory validation represents an important limitation in the current literature. Variations in sample handling, extraction conditions, analytical platforms, and data normalization approaches across research groups can produce substantial discrepancies in reported metabolite composition and bioactivity results, highlight the need for standardized protocols, reference materials, and coordinated multi-laboratory studies.

### 7.3. Regulatory Aspects, Safety Assessment, and Quality Control Frameworks

The regulatory framework governing GP-derived ingredients remains fragmented and, in many cases, insufficiently defined for emerging food and nutraceutical applications. Depending on the form of the ingredient (whole pomace flour, purified extract, or concentrated phenolic fraction), GP-derived products may fall under different regulatory categories, including food ingredients, food additives, dietary supplements, or novel foods. In the European Union, the commercialization of highly concentrated GP extracts may require authorization under the EU Novel Food Regulation (Regulation (EU) 2015/2283) [124] and EFSA [125], whereas in the United States safety evaluation is often pursued through the GRAS pathway. Generally Recognized as Safe (GRAS); (Final Rule) [126]. These procedures require substantial scientific documentation, including compositional characterization, toxicological evaluation, and estimated dietary exposure levels.

A key regulatory challenge arises from the compositional variability of GP, which depends on grape variety, geographic origin, agricultural practices, and winemaking technologies. Such variability complicates the establishment of standardized specifications required for regulatory approval. For example, concentrations of polyphenols, tannins, and residual ethanol may vary considerably between batches, necessitating robust quality control protocols and validated analytical methods. Furthermore, potential contaminants—including pesticide residues, heavy metals, and mycotoxins—must be systematically monitored, particularly when GP is sourced from multiple producers or regions.

Comprehensive safety assessment is also required to evaluate possible physiological interactions associated with high levels of dietary polyphenols and fiber. These include potential effects on mineral absorption, interactions with drug-metabolizing enzymes, and modulation of gut microbiota. While many polyphenols present in GP are naturally occurring dietary compounds, concentrated extracts may result in intake levels significantly higher than those typically achieved through conventional diets. Therefore, toxicological studies, allergenicity assessments, and long-term exposure evaluations may be necessary for regulatory approval, particularly for nutraceutical products or functional foods.

From an industrial perspective, the development of standardized GP-derived ingredients requires the implementation of good manufacturing practices (GMP), traceability systems, and validated quality assurance procedures. These include batch-to-batch consistency testing, stability studies, and specification limits for key bioactive compounds. Establishing harmonized regulatory guidelines and safety thresholds across jurisdictions would facilitate market entry and reduce uncertainty for manufacturers. Clearer regulatory pathways would also support the responsible development of GP-based functional ingredients while ensuring consumer safety and product transparency.

### 7.4. Industrial Valorization, Economic Feasibility, and Circular Economy Integration

Although GP is generated in large quantities as a by-product of winemaking, its large-scale integration into food systems remains limited. Globally, millions of tonnes of GP are produced annually, yet a significant proportion is still directed toward low-value uses such as animal feed, composting, or disposal. Transforming GP into higher-value food ingredients or nutraceutical products therefore represents an attractive opportunity within circular bioeconomy strategies. However, several technical, economic, and logistical challenges currently limit broader industrial adoption.

One of the primary constraints relates to industrial scalability of extraction and processing technologies. Many laboratory-scale extraction methods used to recover polyphenols—such as solvent extraction, ultrasound-assisted extraction, or supercritical fluid extraction—may require significant optimization to become economically viable at industrial scale. Large-scale implementation must consider factors such as energy consumption, solvent recovery, equipment investment, and processing throughput. For example, while advanced techniques may achieve high extraction efficiency in laboratory settings, they may involve capital-intensive equipment or high operational costs that limit their competitiveness compared with conventional extraction processes.

Cost considerations are particularly important when GP-derived ingredients compete with established plant extracts or synthetic antioxidants already used in the food industry. Economic feasibility depends on several factors, including raw material availability, transport logistics from wineries to processing facilities, storage requirements, and processing yield. Because GP is produced seasonally and contains high moisture content, stabilization steps such as drying or ensiling are often required to prevent microbial degradation and preserve phenolic compounds. These additional processing steps increase production costs and must be carefully optimized to ensure economic viability.

Quality control and standardization represent another major challenge for industrial applications. The chemical composition of GP can vary widely depending on grape cultivar, climate conditions, vineyard management practices, and vinification methods. Such variability affects the concentration and profile of phenolic compounds, fiber fractions, and lipid components, potentially influencing both functional properties and biological activity. For industrial use, standardized extraction protocols and well-defined product specifications are therefore required to ensure consistent ingredient quality. Advanced analytical techniques, including chromatographic and spectrometric methods, are increasingly employed to characterize phenolic profiles and establish quality markers for GP-derived ingredients.

Despite these challenges, GP valorization offers significant opportunities within circular economy frameworks. Sequential or integrated biorefinery approaches can enable the recovery of multiple value-added fractions from the same raw material, including polyphenol-rich extracts, dietary fiber ingredients, and seed oils. Such cascading utilization strategies improve resource efficiency and enhance the overall economic value of the by-product. In addition, the incorporation of GP-derived ingredients into food formulations—such as bakery products, beverages, and functional snacks—allows researchers to investigate realistic dietary intake levels and matrix effects on polyphenol bioavailability. Importantly, the sustainability benefits of GP valorization must be evaluated alongside technical and economic feasibility. Life cycle assessment studies suggest that converting winery residues into functional food ingredients can reduce environmental impacts associated with waste disposal while creating additional revenue streams for the wine sector. Nevertheless, successful industrial implementation will depend on the development of scalable processing technologies, well-defined regulatory frameworks, and reliable quality control systems. Addressing these practical considerations is essential to translate the scientific potential of GP-derived metabolites into commercially viable and sustainable food applications.

### 7.5. Bridging Preclinical Mechanisms with Real-World Efficacy

Despite substantial progress in elucidating the biological mechanisms of action of GP bioactives, a clear gap remains in establishing a cause–effect relationship between intake and health outcomes in humans, as required for EFSA and FDA health-claim substantiation. While mechanistic insights derived from in vitro and animal models—including redox homeostasis, inflammatory modulation, and microbiota-mediated metabolism—are increasingly robust, their relevance at achievable dietary intake levels has not been sufficiently demonstrated in human populations. This limitation is compounded by variable bioavailability, metabolic conversion, and interindividual differences in gut microbiota composition, which collectively influence exposure to bioactive metabolites. As highlighted in Section 7.1, future research must prioritize well-controlled human intervention studies that integrate mechanistic endpoints alongside clinically relevant outcomes. The incorporation of systems biology approaches, including targeted and untargeted metabolomics, proteomics, and metagenomics, within randomized controlled trials can support the identification of bioavailable metabolites, biological plausibility, and mechanistic consistency, all of which are critical criteria for regulatory evaluation of health claims.

Furthermore, the adoption of validated, standardized biomarkers of effect—such as established inflammatory mediators, metabolic risk markers, oxidative stress indicators, and gut microbiome signatures—is essential to ensure reproducibility, comparability, and evidence integration across studies. Harmonization of all these will facilitate meta-analyses and weight-of-evidence assessments, thereby strengthening the scientific basis required for regulatory approval, substantiated health claims, and translation of GP-derived bioactives into functional foods and nutraceuticals with demonstrated real-world efficacy.

The consideration of GP as a credible source of fruit-derived secondary metabolites depends on the integration of multidisciplinary research efforts spanning extraction, characterization, biological evaluation, and application. Future progress will require harmonized methodologies, improved understanding of bioavailability and metabolic fate, and the generation of robust human evidence using standardized biomarkers. Equally important is the alignment of GP valorization strategies with sustainable and circular food system models, ensuring that scientific developments translate into environmentally and economically viable applications. By addressing these challenges through coordinated and systems-level approaches, GP-derived bioactives can be more effectively integrated into functional foods and dietary strategies, contributing to a more comprehensive and responsible utilization of fruit secondary metabolites.

## 8. Limitations and Future Perspectives

Despite the growing research and literature supporting the bioactive potential of GP, several important limitations and translational challenges remain insufficiently addressed. One of the primary sources of variability arises from the heterogeneity of raw materials. The chemical composition of GP is highly dependent on grape cultivar, geographical origin, climatic conditions, and viticultural practices, as well as winemaking parameters such as fermentation time and pressing conditions. In addition, the relative proportions of skins, seeds, and stems can differ substantially between batches, leading to marked variability in phenolic profiles, lipid content, and fiber-associated compounds. This intrinsic variability complicates cross-study comparisons and limits the reproducibility and standardization of GP-derived ingredients. Another major limitation concerns the discrepancy between in vitro and in vivo findings. Many studies report strong antioxidant, anti-inflammatory, or antimicrobial effects under controlled in vitro conditions; however, these results often rely on concentrations that are not physiologically achievable through dietary intake. Furthermore, simplified cellular models do not adequately capture the complexity of whole-organism responses, including tissue distribution, metabolic transformation, and interactions with endogenous regulatory systems. As a result, promising mechanistic effects—such as modulation of oxidative stress pathways or inflammatory signaling—may not translate directly into measurable clinical outcomes. Related to this issue are bioavailability constraints and metabolic transformations. A substantial proportion of GP polyphenols, particularly high-molecular-weight proanthocyanidins, exhibit limited absorption in the small intestine and instead undergo extensive biotransformation by the gut microbiota. While these processes can generate bioactive metabolites, they also introduce significant interindividual variability linked to microbiome composition. Consequently, the biological effects of GP cannot be inferred solely from its native composition, but must be considered in the context of digestion, metabolism, and host–microbe interactions. Another critical consideration is the gap between experimental exposure levels and realistic dietary intake. Many studies utilize concentrated extracts or purified compounds at doses that exceed those achievable through conventional food consumption. This raises questions regarding the practical relevance of reported bioactivities, particularly in relation to long-term dietary interventions. More research is needed to establish effective yet realistic intake levels, taking into account food matrix effects, formulation strategies, and consumer acceptability. From a technological perspective, processing and stabilization methods introduce additional layers of variability. Drying, extraction, and storage conditions can significantly alter the stability, bioaccessibility, and functionality of GP-derived compounds. However, these process-related factors are rarely integrated with biological outcome data, limiting the ability to establish clear cause–effect relationships across the valorization chain. Future research should therefore adopt a more integrated and standardized approach, combining well-characterized raw materials, harmonized extraction protocols, and physiologically relevant experimental models. All these efforts will be essential to bridge the gap between promising laboratory findings and practical applications, in this way enabling a more robust assessment of the health potential of GP within food, nutraceutical, and biomedical contexts.

## 9. Conclusions

This review highlights the importance of considering grape pomace valorization within a broader translational framework that connects raw material characteristics with downstream biological effects. From a systems perspective, the potential health relevance of grape pomace-derived compounds can be understood through a sequential pathway that begins with the generation of grape pomace as a by-product of winemaking and continues through several interconnected stages. These include the stabilization and processing of grape pomace, which influence the recovery, transformation, and preservation of bioactive metabolites; the resulting chemical composition of grape-pomace-derived extracts or ingredients, particularly their phenolic and fiber-associated compounds; and the interaction of these metabolites with host biological systems and the gut microbiota after dietary intake. Through metabolic transformations and host–microbe interactions, these compounds may modulate biological processes such as oxidative stress responses, inflammatory signaling, and metabolic regulation, ultimately contributing to potential physiological effects. Future progress in this field will require more standardized characterization of grape pomace, improved understanding of bioavailability and metabolite fate, and well-designed human studies that reflect realistic intake scenarios.

## Figures and Tables

**Table 1:** Recent review articles on grape pomace and their main focus relative to the present review.

Primary Scope	Key Contributions	Limitations/Gaps	Positioning Relative to the Present Review	Reference (Title)
Bioactivity, health benefits, and food applications of GP polyphenols	Comprehensive synthesis of antioxidant, anti-inflammatory, cardioprotective, and antimicrobial effects; discusses incorporation into functional foods	Limited integration of molecular signaling pathways (e.g., Nrf2, NF-κB); minimal discussion of processing–bioactivity relationships and scalability	Focuses on health-promoting effects and food applications; the present review extends this by integrating pathway-level mechanisms with processing and translational considerations	[9]
Sustainable utilization, green extraction, circular economy	Detailed overview of eco-friendly extraction (e.g., supercritical CO_2_, ultrasound-assisted extraction), waste valorization, and industrial sustainability strategies	Less emphasis on biological mechanisms, bioavailability, and clinical relevance of GP compounds	Strong sustainability focus; the present review complements this by linking extraction/process variables with molecular bioactivity and physiological outcomes	[6,10,15]
Phenolic composition, metabolism, pharmacokinetics	In-depth discussion of gastrointestinal digestion, microbial biotransformation, and metabolite distribution; highlights role of gut microbiota	Narrower scope regarding industrial processing, formulation strategies, and functional applications	Provides detailed metabolic insight; the present review expands by integrating metabolism with processing technologies and functional/clinical translation	[3]
Composition, extraction, bioactivity, therapeutic potential	Broad and integrative coverage including extraction techniques, antioxidant activity, and therapeutic potential (e.g., metabolic syndrome, cancer)	Limited mechanistic linkage between processing conditions, compound stability, and pathway-specific effects	Broad overview; the present review adds mechanistic depth and connects processing-induced variability with bioaccessibility and efficacy	[10,12]
Phenolic compounds, extraction, health benefits, applications	Updated summary of phenolic profiles (flavonoids, phenolic acids), industrial uses (food, cosmetics), and health effects	Less structured around signaling pathways and lacks integration of processing–bioactivity–bioavailability continuum	Provides updated overview; the present review emphasizes mechanistic pathways and systems-level integration	[5]
Functional foods, nutraceuticals, product development	Strong focus on incorporation into bakery, dairy, and nutraceutical products; discusses sensory and nutritional enhancement	Limited exploration of molecular mechanisms and clinical validation; minimal discussion of compound stability during processing	Application-oriented; the present review bridges application with mechanistic and translational evidence	[5,7,9]
Recovery technologies, bioactives, industrial applications	Highlights integrated biorefinery approaches and multi-product valorization strategies	Less focus on biological mechanisms and in vivo relevance	Supports processing/valorization perspective; present review integrates these with pathway-level bioactivity	[6,10,15]
Comprehensive valorization, composition, applications	Early framework for circular use of GP including food, feed, and bioenergy	Outdated in terms of omics and mechanistic insights	Provides foundational context; present review builds on this with modern mechanistic and translational advances	[7,9]

**Table 2:** Synergies among metabolite classes.

Interaction Type	Example Compounds	Main Biological Outcome	References
Lipids + Phenolics	Linoleic acid + catechin	↑ Nrf2 activation; ↑ antioxidant defence	[2,3,9,12]
Phenolics + Volatiles	Gallic acid + linalool	Strong antimicrobial synergy	[5,9,13,14]
Lipids + Volatiles	Linoleic acid + β-caryophyllene	↑ Anti-inflammatory effects via NF-κB suppression	[9,12,13]
Phenolics + Lipids	Resveratrol + PUFAs	Stabilized lipids, prolonged antioxidant cycling	[2,3,9,12]
Phenolics + Volatiles + Lipids	Proanthocyanidins + PUFAs + terpenes	Improved gut microbiota modulation, metabolic regulation	[3,9,12,15]

**Abbreviations**: ↑—level increased.

**Table 3:** Semi-quantitative overview of major metabolite classes reported in grape pomace (GP), with representative compounds, concentration ranges, and key sources of variability.

Compound Class	Representative Compounds	Reported Range/Representative Values	Key Variability Factors	Main Relevance	References
Lipids and related compounds	Linoleic acid, oleic acid, tocopherols, sterols	Total lipids: ~3–7% DW; PUFA ~63%, MUFA ~20%, SFA ~17%; linoleic acid in seed oil ~68–78 g/100 g oil	Seed content, cultivar, extraction method	Nutritional lipids; stability; bioavailability effects	[7,10,12]
Total phenolics/ polyphenols	Phenolic acids, flavan-3-ols, flavonols, anthocyanins, stilbenes	Often ~5–10% DW; total phenols in some seed extracts up to ~3351 mg GAE/100 g DW	Pomace fraction, cultivar, vinification, extraction method	Main source of antioxidant-related bioactivities	[9,10,42]
Flavan-3-ols and proanthocyanidins	Catechin, epicatechin, epicatechin gallate, procyanidins	Catechin ~203.6 µg/g DW; epicatechin ~162.5 µg/g DW; epicatechin gallate ~54.6 µg/g DW; procyanidins ~3.6–87.9 µg/g DW	Seed proportion, polymerization degree, extraction conditions	Antioxidant, anti-inflammatory, antiplatelet, antiproliferative potential	[20,21]
Anthocyanins (mainly red GP skins)	Malvidin, petunidin, peonidin, delphinidin, cyanidin derivatives	~131 to 488.8 ± 5.8 mg/100 g dry pomace	Grape variety, skin fraction, vinification, drying	Pigments; antioxidant activity; food-colorant potential	[9,12,42]
Stilbenes	trans-Resveratrol, piceid, viniferins	Usually lower than flavan-3-ols; often qualitative or at low levels	Cultivar, stress conditions, analytical method	Additional antioxidant and health-promoting potential	[12,42]
Volatile organic metabolites (VOMs)	Alcohols, esters, aldehydes, terpenes, volatile phenols	One study identified 52 VOMs; ~38.8% alcohols, ~29.3% carbonyls, ~24.2% esters	Cultivar, fermentation, drying, analytical platform	Aroma, flavor, fragrance; possible bioactivity synergy	[13,14]

**Abbreviations**: GP, grape pomace; DW, dry weight; GAE, gallic acid equivalents; PUFA, polyunsaturated fatty acids; MUFA, monounsaturated fatty acids; SFA, saturated fatty acids; VOMs, volatile organic metabolites.

**Table 4:** Biological activities of GP-derived metabolites, including bioactive fractions, main effects, models used.

Bioactive Fraction/ Compound	Biological Activity	Experimental Model	Key Findings	Reference
Polyphenolic extracts (flavan-3-ols, proanthocyanidins)	Antioxidant, redox modulating	Chemical assays (DPPH, ABTS), in vitro (Caco-2, SW620), in vivo (mice)	Scavenges ROS, reduces lipid peroxidation, enhances SOD, CAT, GPx	[82]
Hydroxylated flavonoids (quercetin, catechin)	Antioxidant	in vitro chemical assays	High radical scavenging due to hydroxylation and polymerization	[83]
Polyphenolic fractions (anthocyanins, flavonoids)	Anti-inflammatory	in vitro (RAW 264.7 cells), in vivo (carrageenan-induced paw edema in mice)	Reduces TNF-α, IL-6, IL-1β, COX-2; inhibits NF-κB	[84]
Phenolic acids (gallic, caffeic)	Antimicrobial	in vitro (*E. coli*, *L. monocytogenes*, *B. megaterium*)	Inhibits bacterial growth; activity enhanced after simulated digestion	[85,86]
Triterpenoids (oleanolic acid)	Antidiabetic	in vitro (muscle cells), in vivo (diabetic rats)	Inhibits α-glucosidase, improves glucose uptake	[87]
GP polyphenolic extracts	Anticancer, antimutagenic	in vitro (Caco-2, SW620)	Inhibits proliferation, induces apoptosis, reduces DNA damage	[88]
Fermented GP fractions	Antioxidant & anticancer	in vitro, in vivo	Enhanced bioavailability; stronger antiproliferative activity	[82,89]

**Table 5:** Bioprocessing and Biotransformation Approaches for Grape Pomace.

Bioprocessing Method	Enzymes/ Microorganisms Used	Main Metabolites Generated or Released	Key Bioactivities Enhanced	Reference
Enzymatic hydrolysis (pectinase, cellulase, hemicellulase)	Pectinases, cellulases, hemicellulases	Anthocyanins, flavan-3-ols, phenolic acids; reduction in proanthocyanidin polymerization	↑ Antioxidant; ↑ Anti-inflammatory; ↑ Antimicrobial	[90,91]
Enzymatic tannin hydrolysis (tannase)	Tannase	Gallic acid, catechin derivatives, de-galloylated tannins	↑ Antioxidant; ↑ Antimutagenic	[92]
β-glucosidase treatment	β-Glucosidase	Aglycones of flavonoids (quercetin, kaempferol), improved release of bound phenolics	↑ Bioavailability; ↑ Cellular antioxidant activity	[93]
Yeast fermentation (SSF or SmF)	*Saccharomyces cerevisiae* (and other yeasts)	Catechin, epicatechin, resveratrol derivatives; increased low-molecular-weight phenolics	↑ Antioxidant; ↑ Anti-inflammatory; technological improvements	[94]
LAB fermentation	*Lactobacillus/Lactiplantibacillus plantarum*, *L. casei*, *L. rhamnosus*	Hydroxycinnamic acids, flavonoid aglycones, phenolic acids	↑ Antidiabetic (α-glucosidase inhibition); ↑ Antioxidant; ↑ Gut bio accessibility	[94]
Fungal fermentation (solid-state)	*Aspergillus niger*, *Rhizopus* spp., other fungi	Protocatechuic acid, gallic acid, catechin, low-DP procyanidins	↑ Antioxidant; ↑ Anticancer; ↑ Antimicrobial	[95,96]
Mixed microbial consortia fermentation (yeast + LAB)	Yeasts + LAB consortia	Simplified phenolic profile; esterase and decarboxylase metabolites	↑ Overall bioactivity spectrum; ↑ Digestive bio accessibility	[97]

**Abbreviations**: ↑—level increased.

**Table 6:** Safety Risk Ranking and Regulatory Considerations for Grape Pomace (GP).

Risk Domain	Realistic Risk Ranking	Key Concerns	Regulatory Pathway—Whole GP Powder (Food/Feed)	Regulatory Pathway—Standardized Extract (Novel Food/Nutraceutical)	References
Contaminants (heavy metals, pesticide residues)	Highest	Accumulation of Cu, Zn, Pb, Cd, Mn, Ni, As; pesticide residues	Compliance with food/feed safety limits; routine screening; supply-chain control; processing (washing, detoxification)	Standardized testing and specification; regulatory dossier demonstrating safe intake levels	[6,10,12]
Mycotoxins & microbial hazards	High	Ochratoxin A, aflatoxins, zearalenone; molds from poor storage	Good agricultural practices; rapid drying/ensiling; monitoring; detoxification steps	Preclinical safety testing; specification of maximum mycotoxin levels; processing validation	[5,10,81]
Anti-nutritional factors & tannins	Moderate	Reduced protein digestibility, mineral chelation; high polyphenol content	Optimization of inclusion rates; processing (enzymatic hydrolysis, fermentation)	Standardization of polyphenol content; evaluation of digestibility and nutrient interactions	[2,3,105]
Drug–nutrient interactions & systemic effects	Low-Moderate	Polyphenols affecting phase I/II enzymes, transporters, platelet function, estrogenic activity	Guidance for maximum inclusion; monitoring of bioactive content	Toxicology studies; evaluation of systemic exposure; risk assessment for health claims	[2,3,23]
Allergenicity & sensory acceptability	Lowest	Residual proteins; color, astringency, off-flavors	Allergen labeling and risk assessment; sensory evaluation	Allergen risk assessment; clinical safety evaluation for concentrated products	[7,42,52]

**Table 7:** Summary of grape pomace (GP) potential applications, formulation examples, benefits and key references (selected).

Application	Formulation/ Typical Inclusion	Primary Benefits/ Outcomes	Representative References	Commercial Status
Fortified bakery (bread, crackers)	GP flour 2–10% (*w*/*w*)	↑ Dietary fiber; ↑ antioxidant capacity; improved nutritional label	[110]	Experimental/Limited commercial adoption
Dairy/Yogurt	GP powder 1–5% or fermented GP preparations	↑ Polyphenols; improved antioxidant status; potential probiotic synergy	[117]	Experimental/Pilot-scale trials
Meat products (natural antioxidant)	GP extract 0.1–1% (antioxidant); GP powder up to 5%	Reduced lipid oxidation; prolonged shelf life	[110]	Commercial in some niche products
Nutraceutical capsules (standardized extract)	100–500 mg extract/day (standardized for TPC or PACs)	Concentrated systemic delivery of polyphenols (antioxidant, metabolic)	[112]	Commercial/Marketed products exist
Animal feed (ruminants)	Dried GP 5–15% of ration (species dependent)	Improved meat/milk antioxidant profile; better fatty acid composition	[111]	Experimental/Limited adoption
Animal feed (poultry/piglets)	GP 2–6% or encapsulated extracts	Improved product oxidative stability; variable performance on growth	[115]	Experimental
Food safety aid (mycotoxin adsorption in foods)	GP husks/extracts added to cereal matrices/bio-sorbents	Reduces mycotoxin bio-accessibility in vitro and in vivo (pig model)	[115]	Experimental/Research stage
Cosmetic/topical formulations	GP extracts or oils in creams (0.1–2%)	Antioxidant, UV-protective claims (in vitro)	[116]	Experimental/Some niche cosmetic products

**Abbreviations**: ↑—level increased.

## Data Availability

No new data were created or analyzed in this study. Data sharing is not applicable to this article.
